# Post-Translational Modifications in Mammalian Folliculogenesis and Ovarian Pathologies

**DOI:** 10.3390/cells14161292

**Published:** 2025-08-20

**Authors:** Dake Chen, Yue Feng, Junjing Wu, Jiawei Zhou, Zipeng Li, Mu Qiao, Tong Chen, Zhong Xu, Xianwen Peng, Shuqi Mei

**Affiliations:** Hubei Key Laboratory of Animal Embryo and Molecular Breeding, Institute of Animal Husbandry and Veterinary, Hubei Academy of Agricultural Sciences, Wuhan 430064, China; chendake@hbaas.ac.cn (D.C.);

**Keywords:** post-translational modification, follicular development, polycystic ovary syndrome, premature ovarian insufficiency

## Abstract

Post-translational modifications (PTMs) of proteins, as the core mechanism for dynamically regulating follicular development, affect the maintenance of mammalian fertility by precisely coordinating granulosa cell–oocyte interaction, metabolic reprogramming, and epigenetic remodeling. Dysregulation of these modifications directly contributes to major reproductive diseases, including polycystic ovary syndrome (PCOS) and premature ovarian insufficiency (POI). Post-translational modifications regulate follicular development through intricate mechanisms. Thus, this review systematically synthesizes recent advances in PTMs, encompassing traditional ones such as phosphorylation, ubiquitination, and acetylation, alongside emerging modifications including lactylation, SUMOylation, and ISGylation, thereby constructing a more comprehensive PTM landscape of follicular development. Furthermore, this study dissects the molecular interaction networks of these PTMs during follicular activation, maturation, and ovulation, and uncovers the common mechanisms through which PTM dysregulation contributes to pathological conditions, including hyperandrogenism in PCOS and follicular depletion in POI. Finally, this review ultimately provides a theoretical basis for improving livestock reproductive efficiency and precise intervention in clinical ovarian diseases.

## 1. Introduction

### 1.1. Folliculogenesis

Successful ovulation and fertilization rely on the precisely orchestrated process of folliculogenesis, a fundamental biological event in female reproductive physiology [1]. Folliculation initiation begins with arrested oocytes in prophase I (diplotene). These cells exit dormancy by bidirectional signaling with the surrounding granulosa cells (GCs): on the one hand, the oocyte secretes GDF9, BMP15, and other factors regulate granulosa cell differentiation [2]; on the other hand, the granulosa cells maintain meiotic arrest of the oocyte through cGMP and other signaling molecules [3]. This interaction drives the activation of primordial follicles. This hierarchical continuum encompasses morphological and functional transformations from primordial follicle recruitment, through antral formation, to ovulatory maturation [4].

**Figure 1:**
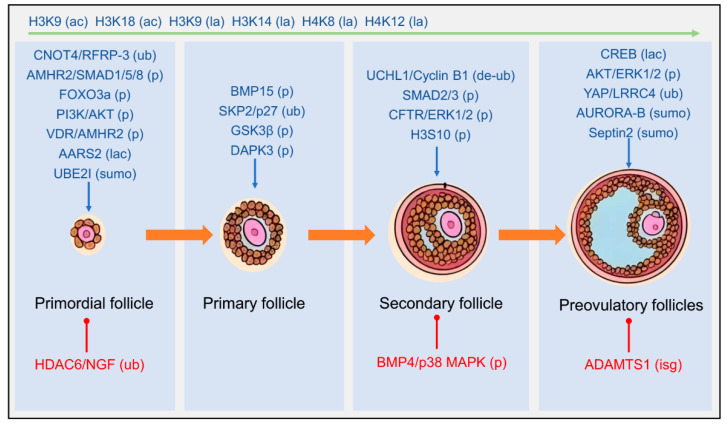
Effects of protein post-translational modifications on follicles at different developmental stages. The process of follicular development is divided into primordial follicles, primary follicles, secondary follicles, and pre-ovulatory follicles, which are distinguished by follicular size as well as the number and structure of granulosa cell layers. At each developmental stage, histone or protein phosphorylation (p), ubiquitination (ub), acetylation (ac), lactylation (la), SUMOylation (sumo), and ISGylation (isg), as listed in the plot, maintain follicular development by regulating granulosa cell proliferation, hormone secretion function, and oocyte meiosis, preventing follicular exhaustion and abnormal follicular development. Blue arrows represent the promotion of follicular development, and red ball-and-stick structures represent the inhibition of follicular development.

Follicles are morphologically classified into four categories, each with distinct functional characteristics [5]. Primordial follicles, quiescent structures consisting of a primary oocyte and flattened granulosa cells, are activated by molecular signals to initiate growth and transition into primary follicles. During this transition, granulosa cells differentiate into a single layer of cuboidal cells. This process is accompanied by oocyte growth and zona pellucida formation [5]. Primary follicles then develop into secondary follicles, which are characterized by multiple granulosa cell layers, early antral clefts, and the recruitment of steroidogenic theca cells. At this stage, the proliferation of granulosa cells depends on cyclin activity regulated by PTM [1,6]. Finally, secondary follicles mature into pre-ovulatory antral follicles. These follicles have a large antrum, an oocyte enclosed within the cumulus oophorus, and increased responsiveness to follicle-stimulating hormone (FSH). This enhanced responsiveness occurs via upregulation of FSH receptor (FSHR) expression, which drives terminal maturation [7]. Different developmental stages correspond to specific histone modifications (such as acetylation (ac), lactylation (lac), etc.) and protein modifications (such as phosphorylation, ubiquitination, etc.). These complex morphological and functional transformations depend on dynamic regulation of key factors via post-translational modifications (PTMs), which form the core focus of this review (Figure 1).

### 1.2. Folliculogenic Dysregulation and Ovarian Diseases

Ovarian disorders frequently arise from reproductive endocrine dysfunction secondary to impaired folliculogenesis, characterized by hallmark pathological features: aberrant primordial follicle activation, arrested antral follicle maturation, and accelerated follicular atresia [8]. These aberrations manifest as distinct clinical entities: premature ovarian insufficiency (POI), polycystic ovary syndrome (PCOS), and other ovarian dysgenesis. Recent studies reveal that PTMs dynamically regulate the activity, stability, and interaction networks of key proteins, directly participating in molecular pathogenesis [9,10].

The PCOS is an important cause of both menstrual irregularity and androgen excess in women. PCOS can be readily diagnosed when women present with the classic features of hirsutism, irregular menstrual cycles, and polycystic ovarian morphology on transvaginal ultrasound (TVUS). In PCOS, PTM-mediated signaling perturbations are central to drive key pathological features, such as insulin resistance [11]; hyperphosphorylation of IRS-1 impairs PI3K/Akt signaling, inducing GC insulin resistance [12]. Constitutive phosphorylation of luteinizing hormone (LH) receptors aberrantly activates the cAMP-PKA cascade, exacerbating hyperandrogenemia and anovulation [13]. Dysregulated histone modifications (e.g., diminished H3K9 acetylation) further disrupt transcription of androgen-synthesizing enzymes [9,14]. Concurrently, imbalanced p53 phosphorylation, potentially driven by metabolic or oxidative stress, promotes granulosa cell apoptosis and contributes to follicular arrest (Table 1) [8,12].

POI is defined as the development of hypergonadotropic hypogonadism before the age of 40 years. The presenting symptoms are similar to those of menopause. In its fully developed form, it is associated with oligomenorrhea or amenorrhea, symptoms of estrogen deficiency, and gonadotropin levels in the menopausal range before age 40 years. In POI, defective PTMs accelerate follicular depletion, thereby reducing ovarian reserve. Mitochondrial protein succinylation, mediated by regulators like SIRT5, influences oocyte quality through metabolic reprogramming and impacts redox balance [15,16], while aberrant modifications of nuclear transport proteins disrupt signaling cascades, thereby affecting the process of oocyte meiosis and fertilization ability [15,16]. Oxidative stress-associated PTMs (e.g., protein carbonylation), as indicated by studies on crystallins and other targets, not only result from but also exacerbate mitochondrial dysfunction and cellular damage, creating a vicious cycle that critically contributes to ovarian aging and follicle loss [16].

Recently, emerging PTMs such as lactylation, SUMOylation, and ISGylation have recently been implicated in regulating ovarian metabolic and signaling networks [9,17], offering new mechanistic insights into disease pathogenesis. Elucidating the functions of these novel PTMs holds significant promise for uncovering diagnostic biomarkers and therapeutic targets. This review will systematically integrate the core associations between PTM aberrations and ovarian disorders, focusing on their general roles in driving pathological features of PCOS, POI, and ovarian dysgenesis, while highlighting recent advances in targeted interventions [9,17].

## 2. Phosphorylation

**Figure 2:**
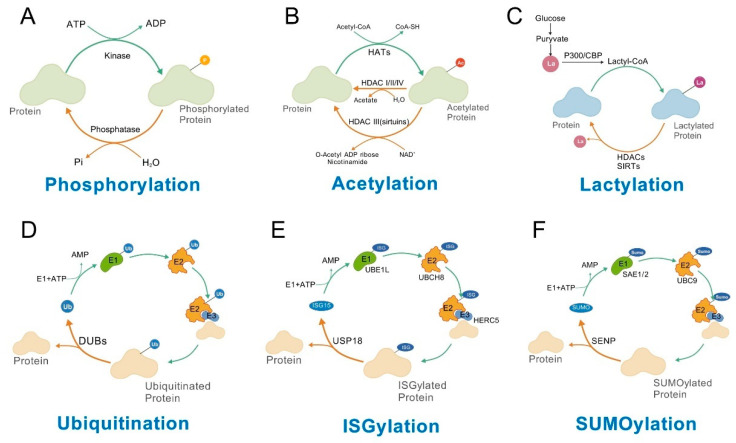
The main modification mechanisms of phosphorylation, acetylation, lactylation, ubiquitination, ISGylation, and SUMOylation. (**A**) Phosphorylation is primarily a process mediated by kinases, which transfer a phosphate group from ATP to substrate proteins. This process can be reversed by phosphatases. (**B**) Acetylation is a process mediated by histone acetyltransferases (HATs) with the involvement of acetyl-CoA, transferring an acetyl group to lysine residues on substrate proteins. Deacetylation is mainly mediated by the HDAC family, which removes the acetyl group from acetylated proteins. Specifically, HDAC classes I, II, and IV utilize H_2_O to hydrolyze the acetyl group, while the HDAC class III family (Sirtuins) employs NAD+ as a cofactor to remove the acetyl group. (**C**) Lactylation is a process that modifies substrate proteins using lactate derived from intracellular uptake or glucose metabolism. Current research primarily indicates that lactate can be converted to lactoyl-CoA via P300/CBP, facilitating the lactylation of substrate proteins. Similarly, delactylation is mainly mediated by the HDAC and SIRT families. (**D**–**F**) Ubiquitination, ISGylation, and SUMOylation are all enzymatic cascade reactions involving E1 activating enzymes, E2 conjugating enzymes, and E3 ligases. The key differences lie in the specific E1/E2/E3 enzymes and the conjugated modifier groups (ubiquitin, ISG15, or SUMO). Additionally, removal of ubiquitination is primarily performed by deubiquitinating enzymes (DUBs), while removal of ISGylation is mainly mediated by USP18, and removal of SUMOylation is predominantly carried out by SENP proteases.

Phosphorylation is the most extensively studied post-translational modification in folliculogenesis, which coordinates granulosa cell–oocyte interactions via a dynamically reversible signaling network, acting as a core regulator of homeostasis across follicular stages from primordial development to ovulation [18]. Phosphorylation is primarily a process mediated by kinases, in which kinases transfer the phosphate group from adenosine triphosphate (ATP) to substrate proteins. On the other hand, this modification can be reversed by phosphatases via removing the phosphate group (Figure 2A). This regulation is first manifested in the precise control of granulosa cell fate: the proliferation, apoptosis, and hormone secretion of granulosa cells all depend on the balance of phosphorylation signals.

**Figure 3:**
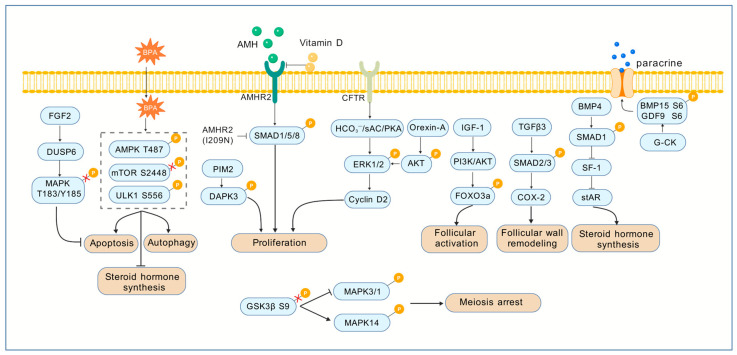
The mechanisms and roles of protein phosphorylation in follicular development. In this figure, BPA perturbs phosphorylation balance of AMPK Thr487, mTOR Ser2448, and ULK1 Ser556, inducing granulosa cell autophagy, apoptosis, and reduced steroidogenesis. AMH transmits signals via AMHR2-mediated phosphorylation of SMAD1/5/8; abnormalities (e.g., I209N mutation) or vitamin D regulation affect follicle reserve. Reduced CFTR expression blocks the HCO_3_^−^/sAC/PKA pathway, decreasing ERK1/2 phosphorylation and cyclin D2 expression to inhibit granulosa cell proliferation. Conversely, PIM2-mediated phosphorylation of DAPK3 enhances cell survival. BMP15 Ser6 phosphorylation by Golgi casein kinase strengthens receptor binding, providing paracrine support for early follicular development. In bovine oocytes, GSK3β Ser9 phosphorylation (inactive form) reduces phospho-MAPK3/1 while maintaining high phospho-MAPK14, impairing meiotic progression. MAPK and SMAD phosphorylation cascades are central to follicular remodeling and fate determination. FGF2-induced DUSP6 dephosphorylates MAPK8 (JNK) Thr183/Tyr185 to inhibit apoptosis, whereas Orexin-A promotes granulosa cell proliferation via AKT Thr308 and ERK1/2 Thr202/Tyr204 phosphorylation. TGFβ3-mediated SMAD2/3 phosphorylation induces COX-2 expression for follicular wall remodeling during ovulation, while BMP-4 reduces StAR expression through SMAD1 phosphorylation and SF-1 inhibition. Energy metabolism-related pathways include the AMPK/mTOR/ULK1 axis in BPA-induced autophagy and IGF-1-activated PI3K/AKT signaling regulating primordial follicle activation via FOXO3a phosphorylation. Collectively, these phosphorylated proteins form a multilevel network maintaining dynamic balance in follicular development, with dysregulation leading to functional abnormalities. p means phosphorylation. The red cross means de-modification.

For instance, AMHR2 transmits AMH signals by mediating the phosphorylation of SMAD1/5/8, and any disruption of this process due to mutations (e.g., the I209N mutation) directly leads to functional abnormalities in granulosa cells [19]. Conversely, environmental disruptors like bisphenol A (BPA) perturb the phosphorylation balance of AMPK Thr487, mTOR Ser2448, and ULK1 Ser556, inducing autophagy and apoptosis in granulosa cells and ultimately reducing the secretion of estradiol, progesterone, and other hormones [20]. In parallel, reduced CFTR expression blocks the HCO_3_^−^/sAC/PKA pathway, decreasing ERK1/2 phosphorylation and inhibiting cyclin D2 expression, thereby impeding granulosa cell proliferation [21]. In contrast, phosphorylation of DAPK3 by PIM2 kinase enhances cell survival, collectively revealing the core principle that “dysregulation equals dysfunction” in phosphorylation signaling (Figure 3) [22].

The maturation of oocytes and their functional coordination with granulosa cells also rely on the precise regulation of phosphorylation. Phosphorylation of the Ser6 site of BMP15 by Golgi casein kinase enhances its binding affinity to receptors, providing paracrine support for early follicular development [23]. In bovine oocytes, the phosphorylation status of GSK3β at Ser9 (inactive form) reduces phospho-MAPK3/1 level and maintained phosphoMAPK14 at a higher level, which could hamper the normal meiosis progression (Figure 3) [24].

Furthermore, phosphorylation forms functional networks by linking key signaling pathways, supporting multiple stages of follicular development. Phosphorylation cascades of the MAPK and SMAD families are central to follicular wall remodeling and cell fate determination: FGF2-induced DUSP6 inhibits the pro-apoptotic activity of MAPK8 (JNK) by dephosphorylating its Thr183/Tyr185 sites [25], while Orexin-A promotes GCs proliferation by activating the phosphorylation of AKT and ERK1/2 at Thr202/Tyr204, with both pathways regulating GCs survival in opposing directions [26]. Meanwhile, TGFβ3-mediated phosphorylation of SMAD2/3 induces COX-2 expression to support follicular wall remodeling during ovulation [27], whereas BMP-4 reduces StAR expression by inducing SMAD1 phosphorylation and inhibiting the transcriptional activity of SF-1 [28]. Phosphorylation pathways related to energy metabolism are equally indispensable: the AMPK/mTOR/ULK1 pathway is central to BPA-induced autophagy in granulosa cells [20]; the PI3K/AKT pathway, activated by IGF-1, regulates primordial follicle activation by modulating FOXO3a phosphorylation [29,30,31]; and vitamin D maintains the integrity of AMH signaling by reducing AMHR2 level and phosphorylation of SMAD1/5/8, complementing AGEs-induced abnormal AMH signaling pathway in protecting primordial follicle reserves [32]. Above all, these phosphorylated proteins form a multi-level regulatory network of phosphorylation signaling in follicular development (Figure 3).

**Figure 4:**
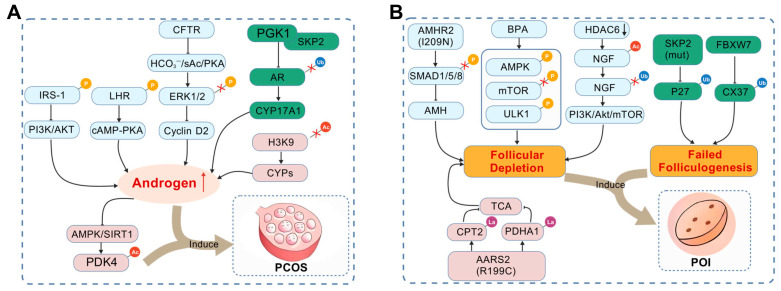
Mechanistic pathway map of post-translational modifications regulating PCOS and POI. (**A**) In polycystic ovary syndrome (PCOS), key pathogenic pathways involve dysregulated PTMs. Phosphorylation-related perturbations include hyperphosphorylation of IRS-1, which impairs PI3K/Akt signaling and induces insulin resistance in granulosa cells (GCs); constitutive phosphorylation of luteinizing hormone (LH) receptors aberrantly activates the cAMP-PKA cascade, exacerbating hyperandrogenemia and anovulation; and reduced CFTR expression blocks the HCO_3_^−^/sAC/PKA pathway, decreasing ERK1/2 phosphorylation and inhibiting cyclin D2 expression, thereby arresting granulosa cell proliferation and follicle development. Ubiquitination dysfunction is characterized by abnormally elevated phosphoglycerate kinase 1 (PGK1), which binds to the E3 ubiquitin ligase Skp2 and inhibits its activity, preventing ubiquitination and degradation of the androgen receptor (AR), leading to AR accumulation, increased local ovarian androgen synthesis via CYP17A1 activation, and chronic inflammatory microenvironment formation. Acetylation dysregulation involves hyperandrogen-induced inhibition of the AMPK/SIRT1 axis, elevating PDK4 acetylation and disrupting granulosa cell glycolysis, along with diminished H3K9 acetylation that impairs transcription of androgen-synthesizing enzymes. The red upward arrow means the increased androgen level. (**B**) In premature ovarian insufficiency (POI), critical pathogenic mechanisms are linked to defective PTMs. Phosphorylation abnormalities include the I209N mutation of AMHR2, which blocks SMAD1/5/8 phosphorylation, interrupts anti-Müllerian hormone (AMH) signaling, and impairs primordial follicle reserve maintenance; bisphenol A (BPA) activates AMPK (Thr487), inhibits mTOR (Ser2448), and activates ULK1 (Ser556), inducing granulosa cell autophagy and accelerating follicle reserve depletion. Ubiquitination dysfunction involves reduced HDAC6, which leads to insufficient deacetylation of nerve growth factor (NGF), impairing its ubiquitination and degradation, thereby over-activating the PI3K/Akt/mTOR pathway and causing excessive primordial follicle activation; Skp2 deficiency hinders ubiquitination and degradation of p27, inhibiting granulosa cell transition from G1 to S phase and impairing proliferation; FBXW7 mutation reduces ubiquitination efficiency of connexin 37, disrupting oocyte–granulosa cell communication. UBE2I deficiency downregulates maternal effect genes and disrupts zygotic genome activation, leading to follicle depletion. Lactylation dysregulation involves AARS2 (R199C mutation) increasing lactylation levels of downstream proteins and inhibiting CPT2-mediated fatty acid oxidation and PDHA1-driven pyruvate entry into the tricarboxylic acid cycle, thus accelerating follicle recruitment and depletion. p means phosphorylation; Ub means ubiquitination; Ac means acetylation; La means lactylation; the red cross means de-modification.

Dysregulation of the phosphorylation network is a core pathological basis for ovarian disease. Its role in both PCOS and POI is related to disease-specific mechanisms as well as common control nodes, which are critical for the discovery of mechanisms and therapy (Figure 4). In PCOS, phosphorylation dysregulation primarily manifests as dual impairments in granulosa cell function and microenvironmental homeostasis. Reduced CFTR expression leads to insufficient ERK1/2 phosphorylation, directly arresting granulosa cell proliferation and contributing to the formation of polycystic follicles [21]. Concurrently, excess androgens suppress AMPK phosphorylation at Thr172, downregulating the SIRT1/PDK4 pathway and disrupting the coordination between endometrial decidualization and follicular microenvironments [33]. These abnormalities jointly demonstrate the multi-pathway synergistic characteristics of phosphorylation dysregulation in PCOS. Among them, the potential abnormality of AKT/ERK phosphorylation in the insulin signaling pathway may further link metabolic disorders to follicular development disorders. However, the specific phosphorylation sites and molecular mechanisms of this association still need to be explored in depth [34]. This also provides a key direction for deciphering the complex pathology of PCOS and developing targeted treatment strategies.

The pathogenesis of POI is closely associated with disruptions in key nodes of phosphorylation cascades. The I209N mutation of AMHR2 blocks SMAD phosphorylation through a dominant-negative effect, interrupting AMH signaling and impairing primordial follicle reserve maintenance [19]. Similarly, BPA exposure accelerates follicle reserve depletion by activating the AMPK/mTOR pathway to induce granulosa cell autophagy—this mechanism echoes AMPK dysregulation in PCOS, forming a cross-disease signaling connection [20]. Notably, targeted intervention in AKT1 phosphorylation at Ser473 (e.g., using the PTK2B inhibitor PF-431396) can improve granulosa cell survival in POI by inhibiting inflammation and apoptosis, offering a promising direction for precision therapy [35].

Importantly, the impact of phosphorylation dysregulation extends to cross-organ/disease associations: abnormal AKT phosphorylation in NKX2-1-positive thyroid cells can indirectly disrupt follicular development through metabolic disorders in thyroid diseases [36]. Additionally, vitamin D deficiency, due to impaired VDR function, causes dysregulated AMHR2 phosphorylation—this mechanism may represent a shared pathological link between PCOS and POI [32]. These findings highlight the central role of phosphorylation signaling in ovarian diseases, which suggests its potential as a diagnostic biomarker and therapeutic target in the future. Restoring the phosphorylation balance at key sites could enable synergistic intervention in multiple ovarian diseases.

## 3. Ubiquitination

Ubiquitination, a conserved post-translational modification mediated by E1-E2-E3 ligases and deubiquitinating enzymes (Figure 2D), centrally regulates ovarian folliculogenesis by modulating protein turnover and signaling. It integrates multi-level signals—pituitary gonadotropins (endocrine), local ovarian GDF9/BMP15 (paracrine), and intracellular metabolic/stress signals [37]—orderly regulating folliculogenesis from primordial quiescence, primary initiation, and secondary growth to maturation and ovulation. This regulation relies on ubiquitin chain diversity: K48-linked polyubiquitination (linear) is recognized by the 26S proteasome, marking proteins for degradation [38]; K63-linked chains (branched) bind signaling molecules, promoting complex assembly and signal amplification to regulate transduction efficiency [38,39].

At the primordial follicle stage, the ubiquitination system maintains the dynamic balance between quiescence and activation through the synergistic action of E3 ligases and deubiquitinating enzymes. For instance, histone deacetylase HDAC6 can enhance the binding ability of nerve growth factor to E3 ligases by catalyzing the deacetylation of NGF, thereby promoting ubiquitination modification [40]. This process ultimately inhibits the PI3K/Akt/mTOR signaling pathway, of which silencing is crucial for maintaining the dormant state of primordial follicles [40]. Under physiological conditions, the expression of HDAC6 is dynamically regulated by factors such as inhibin and activin in the local ovarian microenvironment: it remains highly expressed during the fetal and childhood periods to ensure the stable reserve of the primordial follicle pool, while its expression gradually decreases after puberty to provide conditions for the cyclical activation of follicles. Conversely, abnormal reduction in HDAC6 during aging leads to enhanced stability of NGF, triggering excessive activation of the PI3K/Akt/mTOR pathway, which in turn causes premature recruitment and depletion of primordial follicles [40]. In contrast, the E3 ligase CRL4 maintains the transcription in growing oocytes by targeting MeCP2 for degradation to prevent DNA hypermethylation [41].

During the development of antral follicles, the ubiquitination system further regulates the proliferation and differentiation of GCs. In cell cycle regulation, the E3 ligase Skp2 promotes the transition of GCs from the G1 phase to the S phase by recognizing and mediating ubiquitination and degradation of the cell cycle inhibitor p27, thereby relieving its inhibition on the cyclin E-CDK2 complex [42]; meanwhile, the deubiquitinating enzyme UCHL1 maintains the stability of cyclin B1 by removing its ubiquitin chains, ensuring that GCs successfully complete the G2/M transition of mitosis [43]. The synergistic effect of these two molecules guarantees the orderly proliferation of GCs during follicle growth, providing structural and nutritional support for oocyte maturation.

The precise regulation of ubiquitination is important for oocyte development and meiosis. FBXW7, a component of the SCF E3 ligase complex, can dynamically regulate the permeability of gap junctions by mediating ubiquitination of the gap junction protein connexin 37, thereby controlling the transport of small signaling molecules (such as cAMP and ATP) between oocytes and GCs [44]; UCHL1, on the other hand, maintains the structural integrity of the synaptonemal complex by specifically removing ubiquitin chains from SYCP3, the core protein of the meiotic synaptonemal complex, ensuring the correct pairing and segregation of homologous chromosomes. If UCHL1 is functionally defective, SYCP3 will accumulate ubiquitination and be degraded, leading to abnormal chromosome segregation and oocyte apoptosis [43]. This mechanism is highly conserved in evolution. For example, in medaka fish, the transcription factor foxl3 can activate the expression of the E3 ligase fbxo47, ensuring the stable commitment of germ cells to the oocyte fate [45].

In addition, ubiquitination also affects follicular development by regulating the metabolic homeostasis of GCs. The transcriptional coactivator YAP plays a key role in the metabolic regulation of GCs. LRRC4 can restrict YAP’s nuclear translocation by promoting ubiquitination of YAP; if LRRC4 is deficient, YAP will accumulate in the nucleus and activate the expression of DRP1 (a mitochondrial fission-related protein), leading to excessive mitochondrial fission and impaired oxidative phosphorylation. Eventually, follicular development is arrested due to insufficient energy supply [46].

In summary, the ubiquitination system acts as a “molecular switch” at each stage of folliculogenesis by constructing a multi-level regulatory network, accurately converting extracellular signals into intracellular biological responses, and ensuring the orderly progression of follicles from the primordial stage to mature ovulation.

The imbalance in the ubiquitination system is an important molecular basis for the occurrence and development of various ovarian diseases. It disrupts the normal regulatory network of follicular development, leading to abnormal ovarian function (Figure 4). In PCOS, abnormal ubiquitination of the androgen receptor (AR) is one of the core pathological features. Under physiological conditions, the E3 ligase Skp2 can recognize and mediate ubiquitination and degradation of AR by targeting its degradation signal sequence, thereby maintaining the dynamic balance of AR [14]. However, in PCOS patients, the expression of phosphoglycerate kinase 1 (PGK1) is abnormally elevated. PGK1 can directly bind to Skp2 to inhibit its ubiquitination activity on AR, resulting in enhanced stability of AR protein and its accumulation in GCs [47]. The abnormal activation of AR further initiates a cascade of downstream target gene responses: on the one hand, it continuously activates the expression of CYP17A1, a key enzyme in androgen synthesis, leading to increased local androgen levels in the ovary [48]; on the other hand, it promotes GCs survival by inhibiting pro-apoptotic factors [49]. These factors not only stimulate abnormal proliferation of GCs but also recruit immune cells such as macrophages to infiltrate the ovarian stroma, forming a chronic inflammatory microenvironment. Together, they ultimately hinder the transformation of follicles from the preantral stage to the antral stage, resulting in the accumulation of a large number of small follicles in the ovary and ovulatory dysfunction [14,47].

For POI, oligogenic defects in the ubiquitination regulatory network are important pathogenic mechanisms. Large-scale genomic sequencing studies have shown that approximately 23.5% of POI patients carry pathogenic variants in at least two ubiquitination-related genes. These variants are mainly concentrated in two types of genes: one type consists of key molecules regulating oocyte meiosis (such as FBXW7, UCHL1), and the other type consists of factors involved in maintaining GC function (such as SKP2, LRRC4), suggesting that systemic functional defects in the ubiquitination system are important drivers of POI [50,51]. These pathogenic variants are mostly missense mutations or splice site mutations. For example, the R465H mutation in FBXW7 weakens its binding ability to connexin 37, leading to reduced ubiquitination efficiency of gap junction proteins and disruption of signal transmission between oocytes and GCs; the C152F mutation in UCHL1 destroys its catalytic active center, making it unable to effectively remove ubiquitin chains from SYCP3, resulting in synaptonemal complex disintegration and abnormal chromosome segregation [51]. This oligogenic inheritance pattern shows significant synergistic effects in POI. For instance, the combined deficiency in FBXW7 and SKP2: abnormal FBXW7 function disrupts the communication balance between oocytes and GCs [44], while SKP2 deficiency inhibits GC proliferation due to impaired degradation of p27 [42]. The superimposed effect of these two defects accelerates the depletion of the follicle pool more significantly than a single gene defect, ultimately leading to premature ovarian failure [51].

In conclusion, ubiquitination maintains ovarian functional homeostasis by precisely regulating various links of follicular development, while its dysregulation participates in the occurrence and development of PCOS, POI, and other ovarian diseases by disrupting hormone signals, cell communication, and metabolic balance. In-depth analysis of its mechanisms can provide new theoretical foundations and intervention strategies for the precise treatment of ovarian diseases.

## 4. Acetylation

In the intricate process of folliculogenesis, acetylation modifications exert regulatory effects through a hierarchical network, encompassing gene expression, cellular architecture, and energy homeostasis, thereby governing follicle maturation and oocyte competency [52]. Acetylation is a process mediated by histone acetyltransferases (HATs), which requires the involvement of acetyl-CoA and specifically transfers the acetyl group to the lysine residues of substrate proteins. And deacetylation is mainly mediated by the histone deacetylase (HDAC) family (Figure 2B). Histone acetylation, as a pivotal epigenetic regulator, maintains dynamic equilibrium to mediate chromatin remodeling and transcriptional activation [18,53]. During primordial follicle formation, the transient upregulation of histone H3K9 acetylation (H3K9ac) in primordial germ cells PGCs migrating to the genital ridge is closely associated with the transcriptional activation of germ cell-specific genes such as Ddx4 and Sycp3, laying the molecular foundation for primordial follicle assembly [54]. As follicles enter the growth phase, histone marks including H3K9ac and H3K18ac accumulate progressively with oocyte growth, reaching a peak at the germinal vesicle (GV) stage. By loosening chromatin conformation, these modifications facilitate the expression of meiosis-related genes, ensuring the proper progression of oocytes through meiotic stages [54]. In granulosa cells, FSH triggers synergistic H3S10 phosphorylation and H3K9ac via the protein kinase A (PKA) pathway, activating key estrogen-synthetic genes like CYP19A1, which drives dominant follicle selection and maturation, reflecting the crosstalk between hormonal signaling and epigenetic regulation [18,55]. Beyond histone acetylation, non-histone acetylation plays a crucial role in modulating cytoskeletal integrity and intercellular communication. α-tubulin acetylation at lysine 40 (α-tubulin K40ac) is essential for spindle stability: the acetyltransferase Ikbkap (Elp1) promotes microtubule polymerization through this modification, and its depletion leads to spindle polarity disruption, chromosome misalignment, and reduced oocyte maturation rates (70.9% vs. 92.2% in controls) [56]. In Addition, acetylation-mediated metabolic reprogramming is central to follicle viability, coordinating with intercellular communication to meet stage-specific energy demands. The AMPK/SIRT1 axis modulates granulosa cell function by regulating PDK4, a key glycolytic enzyme: AMPK activation enhances SIRT1 deacetylase activity, which activates PDK4 to inhibit pyruvate dehydrogenase, thereby fueling follicle development [33,57]. Furthermore, the deficiency in α1AMPK reduces the activity of the deacetylase HDAC, leading to a slight increase in histone H3 acetylation at lysine 9 (H3K9) and lysine 14 (H3K14) residues, accompanied by abnormal mitochondrial structures, ultimately reducing the energy reserve of oocytes and impairing embryonic development [57].

Dysregulated acetylation underpins multiple ovarian pathologies, emerging as a promising therapeutic target (Figure 4). In PCOS, follicular hyperandrogenism inhibits the AMPK/SIRT1 axis, elevating PDK4 acetylation and disrupting granulosa cell glycolysis [33]. Reduced SIRT1 activity also increases H3K9ac at profibrotic gene promoters, promoting ovarian fibrosis [33]. Preclinically, SIRT1 agonists (e.g., SRT1720) restore PDK4 deacetylation, while AMPK activators (e.g., A-769662) reverse Cx37 downregulation, improving intercellular communication [33,57]. Oocyte quality decline is often linked to defective α-tubulin acetylation: Ikbkap deletion reduces α-tubulin K40ac, causing spindle abnormalities and aneuploidy (51.5% vs. 10.3% in controls) [56]. Similarly, AMPKα1 depletion lowers SIRT1 levels, increasing H3K9/K14ac and disrupting oocyte gene expression, leading to two-cell stage arrest [57]. Targeting these pathways with α-tubulin acetyltransferase agonists or HDAC6 inhibitors may rescue spindle stability and embryonic viability [56,57]. Furthermore, epigenetic dysregulation of steroidogenic genes contributes to ovarian dysfunction. CYP19, critical for estrogen synthesis, requires H3K9ac enrichment at its PII promoter during the follicular phase and PI.1 promoter during the luteal phase [58]. Reduced H3K9ac at these loci impairs estrogen production and dominant follicle selection, which could be reversed by HAT activators (e.g., p300 agonists) [58].

## 5. Lactylation

Lactylation is a new type of post-translational modification, which is closely correlated with the concentration of lactic acid, thus is involved in metabolism process [59]. Lactylation is a process that modifies substrate proteins using lactate taken up intracellularly or produced by glucose metabolism. Current research mainly indicates that lactate can be converted to lactyl-CoA through E1A-binding protein p300 (P300) or cyclic AMP response element-binding protein (CBP), thereby promoting the lactylation of substrate proteins. Similarly, delactylation is mainly mediated by the HDAC family and the sirtuin (SIRT) family (Figure 2C). As a metabolite-dependent post-translational modification, lactylation plays a critical role in multiple stages of follicular development by integrating cellular metabolism with epigenetic regulation [60]. In GCs, mutations in mitochondrial alanyl-tRNA synthetase 2 (AARS2), notably the R199C variant, elevate lactylation levels [61,62]. This lactylation modification inhibits CPT2-mediated fatty acid oxidation and PDHA1-driven pyruvate entry into the tricarboxylic acid cycle, leading to the accumulation of free fatty acids (FFAs) and glycolytic intermediates [63]. These metabolic changes synergistically activate the PPARγ and mTORC1 signaling pathways in GCs, collectively promoting follicular growth and maturation [62]. During oocyte maturation, histone lactylation exhibits dynamic regulation: markers including H3K9la, H3K14la, H4K8la, and H4K12la are highly expressed at the germinal vesicle (GV) stage, with progressive downregulation as meiosis advances to metaphase II (MII) [64]. Exogenous lactate supplementation (10 mM) enhances these lactylation marks—particularly H3K14la and H4K12la—improving oocyte maturation rates and spindle integrity by upregulating oxidative phosphorylation-related genes [64].

During luteinization, human chorionic gonadotropin induces a hypoxic microenvironment in GCs, stimulating lactate production via lactate dehydrogenase A (LDHA). This lactate pool promotes both histone lactylation (e.g., H3K18la) and non-histone lactylation of cAMP response element-binding protein (CREB) at K136. H3K18la activates the transcription of steroidogenic genes (CYP11A1, STAR) through promoter enrichment, while CREB K136la enhances transcriptional activity, collectively driving progesterone synthesis [65]. Notably, in female germline stem cells, H3K18 lactylation (H3K18la) activates the transcription of YTH domain family 2 (Ythdf2), an N^6^-methyladenosine (m^6^A) reader. Ythdf2 binds m^6^A-modified Ets1 mRNA to promote its degradation, thereby restraining FGSC proliferation, which is crucial for maintaining germline stem cell homeostasis [66].

Dysregulation of lactylation is closely associated with the pathogenesis of various ovarian disorders, primarily through mechanisms involving follicular exhaustion, hormonal synthesis abnormalities, and impaired oocyte quality (Figure 4). In POI, hyperactivity or mutations of AARS2 enhance its lactyltransferase activity, accelerating follicular recruitment and depletion, thereby reducing ovarian reserve [61,62]. Clinical studies confirm elevated serum lactate and FFAs in POI patients, which negatively correlate with the ovarian reserve marker AMH. Importantly, β-alanine-mediated inhibition of lactylation delays follicular exhaustion in murine models, providing a preclinical basis for POI therapeutic intervention [62].

Protein lactylation also plays an important role in some other female diseases and application prospects. In luteal insufficiency, impaired expression of H3K18la or CREB K136la weakens the transcriptional activation of steroidogenic genes, reducing progesterone synthesis. Inhibition of lactylation (e.g., with oxamate or C646) further decreases luteinization markers and corpus luteum formation, exacerbating luteal dysfunction [65]. Additionally, during oocyte preservation, vitrification-induced oxidative stress reduces H4K12la levels, leading to abnormal spindle assembly and impaired ZGA. The walnut-derived peptide TW-7 rescues these defects by reducing reactive oxygen species, restoring LDHA/LDHB activity, and enhancing EP300-mediated H4K12la, thereby improving blastocyst development from vitrified MII oocytes, indicating that H4K12la is a critical epigenetic marker of oocyte quality [67]. Hypoxic conditions (2% O_2_), however, reduce histone lactylation (H3K23la, H3K18la) in pre-implantation embryos, impairing zygotic genome activation and developmental potential, highlighting the oxygen-dependent regulation of lactylation [68].

## 6. SUMOylation and ISGylation

SUMOylation and ISGylation, two prominent ubiquitin-like post-translational modifications (PTMs), share functional parallels in regulating cellular processes such as meiotic progression, transcriptional activity, and stress responses, while exhibiting distinct mechanistic features [52,69]. Similar to ubiquitination, SUMOylation and ISGylation are also enzymatic cascade reactions involving E1 activating enzymes, E2 conjugating enzymes, and E3 ligases. It uses ISG15 (interferon-stimulated gene 15 protein) or SUMO as the conjugated modifier group to substrate proteins (Figure 2E,F). Both modifications are involved in complex regulatory networks that coordinate oocyte competence and follicular development across species, from C. elegans to mammals [52,69].

A key interconnection between them is that SUMOylation can enhance ISG15 conjugation by stabilizing the HERC5 E3 ligase, highlighting the interdependence within the PTM network [70]. In the process of folliculogenesis, SUMOylation and ISGylation exert their regulatory effects through multiple mechanisms. SUMOylation plays a crucial role in ensuring accurate chromosome congression and spindle integrity during meiosis. For instance, in C. elegans, the SUMO E3 ligase GEI-17 mediates the SUMOylation of KLP-19, which is essential for the assembly of the midbivalent ring complex and chromosome alignment [71]. In mammalian oocytes, Aurora-B SUMOylation at Lys207 is necessary for maintaining spindle stability, and mutation of this site leads to chromosome misalignment and metaphase I arrest [72]. Additionally, SUMOylation of Septin2 promotes chromosome congression in mouse oocytes [73], and Polo-like kinase 1 (PLK1) undergoes SUMO-2/3 modification, which is reversed by SENP3 to ensure meiotic transition [74]. These findings, consistent with the conserved role of SUMOylation in spindle organization demonstrated in previous studies [75], and the spatial validation of SUMO-2/3 enrichment at centromeres during mouse oocyte meiosis [76], collectively highlight the significance of SUMOylation in meiotic spindle dynamics and chromosome segregation. SUMOylation also maintains transcriptional silencing in fully grown oocytes, critical for preserving maternal gene stores. Oocyte-specific Ube2i knockout mice show failed chromatin remodeling and premature activation of zygotic genome activation [77,78], while SUMOylation of transcription factors SOHLH1 and NOBOX enhances their DNA-binding activity to promote maternal effect gene expression [79]. Moreover, UBE2I (UBC9) localizes to nuclear speckles to stimulate transcription, linking SUMOylation to RNA processing [76]. In terms of meiotic resumption and metabolic integration, SUMOylation of the Akt/PKB pathway promotes germinal vesicle breakdown [80], and in obese mice, altered responses of ovarian SUMO-2/3 targets to genotoxic stress link SUMOylation to metabolic resilience [81].

ISGylation, on the other hand, functions in antiviral responses and ovarian homeostasis. Viral OTU proteases can disrupt host SUMO/ISGylation, impairing antiviral immunity [82]. In pigs, CREBRF suppresses granulosa cell apoptosis and enhances estradiol synthesis via the ISG15-ISGylation axis [83], while ISG15 knockout in mice increases ovulation rate by reducing ADAMTS1 degradation [84]. In ovulation and steroidogenesis, the ISG15-ADAMTS1 axis negatively regulates ovulation through promoting ADAMTS1 proteasomal degradation [84], whereas ISGylation supports follicle health by promoting E2 synthesis [83]. Additionally, ISGylation mediates ovarian responses to stress, with USP18, an ISG15 deconjugase, balancing ISGylation to maintain reproductive tract homeostasis [85], and post-ovulatory aging being linked to SUMO/ISGylation dysregulation, affecting oocyte quality [86].

Dysregulation of SUMOylation and ISGylation has significant implications for ovarian diseases. In POI, UBE2I deficiency causes follicle depletion in mice, accompanied by downregulated maternal effect genes and misactivation of zygotic genome activation [77,78]. Moreover, ovarian SUMO-2/3 dysregulation in obese mice impairs DNA repair, exacerbating follicle loss [81]. In viral and metabolic ovarian damage, viral OTU proteases disrupt SUMO/ISGylation, enhancing pathogenicity [82]. Obesity-induced ISG15 upregulation in granulosa cells disrupts mitochondrial function, promoting follicular atresia [84]. Additionally, in cisplatin-resistant ovarian cancer, ISG15 modifies hnRNPA2B1 to suppress ABCC2 translation, enhancing drug sensitivity [87]. These findings underscore the importance of maintaining proper SUMOylation and ISGylation levels for ovarian health. Therefore, SUMOylation or ISGylation of proteins such as SENP3 [88] or USP18 [85] could be the important targets for translational research in treating POI or enhancing fertility [52].

## 7. Conclusions

**Figure 5:**
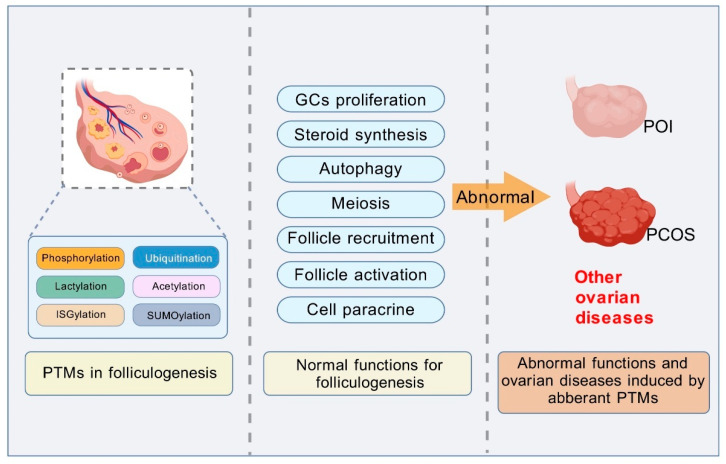
The roles of post-translational modifications (PTMs) in folliculogenesis and ovarian diseases. The PTMs, including phosphorylation, ubiquitination, lactylation, acetylation, ISGylation, and SUMOylation, regulate multiple normal functions during folliculogenesis, such as granulosa cells (GCs) proliferation, steroid synthesis, autophagy, meiosis, follicle recruitment, follicle activation, and cell paracrine interactions. However, when PTMs become aberrant, normal folliculogenesis processes are disrupted. This abnormality, for instance, high androgen, follicular depletion, GCs apoptosis, can induce various ovarian diseases including premature ovarian insufficiency (POI) and polycystic ovary syndrome (PCOS).

Protein post-translational modifications (PTMs) constitute a core regulatory network orchestrating mammalian folliculogenesis, governing processes from primordial follicle activation to ovulatory maturation. This review systematically integrates the roles of PTMs in ovarian physiology and pathology (Table 1), encompassing both classical modifications—phosphorylation, ubiquitination, and acetylation—and emerging ones such as lactylation, SUMOylation, and ISGylation, all of which coordinate granulosa cell–oocyte crosstalk, metabolic reprogramming, and epigenetic remodeling. Among these, the three classical PTMs—phosphorylation, ubiquitination, and acetylation—function, respectively, as key signaling modulators, “molecular switches,” and regulators of epigenetic states and metabolic enzymes. Additionally, emerging PTMs including lactylation, SUMOylation, and ISGylation further expand this regulatory network, with lactylation bridging metabolism and epigenetics, and SUMOylation/ISGylation ensuring meiotic spindle stability and stress responses. These modifications work coordinately, ensuring the GCs and oocytes function well, and jointly promoting the follicular development and maintaining the integral functions of oocytes (Figure 5).

These PTMs do not act in isolation but form interconnected networks. For instance, SUMOylation stabilizes HERC5 to enhance ISGylation, while phosphorylation–ubiquitination crosstalk fine-tunes FOXO3a activity, underscoring that coordinated interactions are critical for maintaining folliculogenesis balance. This interplay also underpins disease pathogenesis: in PCOS, synergistic defects in phosphorylation (IRS-1), ubiquitination (AR), and acetylation (PDK4) drive hyperandrogenism and follicular arrest; in POI, coordinated abnormalities in SUMOylation, ubiquitination, and lactylation accelerate follicle depletion via meiotic dysfunction and metabolic disorders.

Building upon these insights, we propose that future investigations should focus on three key directions to advance the field. First, the single-cell and spatial omics technologies is recommended to dissect the spatiotemporal dynamics of PTMs, thereby elucidating stage-specific and cell-type-specific modification patterns. Second, we suggest decoding the crosstalk mechanisms among PTMs. For example, how phosphorylation and ubiquitination coordinately regulate key proteins, such as SMADs, to reveal network-level regulatory principles. Third, we advocate translating these mechanistic insights into therapeutic strategies, including the development of SIRT1 agonists for PCOS and AARS2 inhibitors for POI. Ultimately, these findings will improve clinical therapy in reproductive disorders and enhance livestock reproductive efficiency.

**Table 1:** The main proteins and their post-translational modifications involved in follicular development and ovarian diseases.

Modification Type	Modified Proteins	Effect	Affected Disease or Function (Phenotype)	Species	References
Phosphorylation	SMAD1/5/8	AMHR2 mutation inhibits SMAD1/5/8 phosphorylation and blocks AMH signaling	POI (impaired primordial follicle reserve)	Human	[19]
	ERK1/2	Reduced CFTR expression blocks the HCO_3_^−^/sAC/PKA pathway and decreases ERK1/2 phosphorylation	PCOS (granulosa cell proliferation arrest, follicle arrest)	Human	[21]
	BMP15 (Ser6)	Golgi casein kinase phosphorylates Ser6, enhancing its binding ability to receptors	Supports early follicular development and oocyte–granulosa cell paracrine signaling	Human	[23]
	GSK3β (Ser9)	Phosphorylation at its Ser9 (inactive form) reduces phospho-MAPK3/1 level and maintained phosphoMAPK14 at a higher level, which could hamper the normal meiosis progression	Regulates spatiotemporal coordination between oocyte meiosis and granulosa cell proliferation	Cattle	[24]
	MAPK8 (JNK, Thr183/Tyr185)	DUSP6 dephosphorylates its Thr183/Tyr185, inhibiting its pro-apoptotic activity	Maintains granulosa cell survival and follicular development	Sheep	[25]
	SMAD2/3 (Ser465/467)	TGFβ3 induces SMAD2/3 phosphorylation, promoting nuclear translocation and regulating COX-2 expression	Supports follicular wall remodeling during ovulation	Human	[27]
	p38 MAPK (Thr180/Tyr182)	BMP-4 inhibits SMAD1 phosphorylation, reducing StAR expression	Balances follicular luteinization and progesterone secretion	Sheep	[28]
	AMPK/mTOR/ULK1 (AMPK Thr487, mTOR Ser2448, ULK1 Ser556)	BPA activates AMPK, inhibits mTOR, and activates ULK1, inducing granulosa cell autophagy	Abnormal follicular development, increased granulosa cell apoptosis in PCOS and POI	Human	[20]
	FOXO3a	IGF-1 activates the PI3K/AKT pathway, regulating FOXO3a phosphorylation	Regulates primordial follicle activation	Pig	[30]
	IRS-1	Hyperphosphorylation of IRS-1 impairs PI3K/Akt signaling	Insulin resistance in granulosa cells in PCOS	Human	[12]
	LH receptor	Constitutive phosphorylation of LH receptor aberrantly activates the cAMP-PKA cascade	Hyperandrogenemia and anovulation in PCOS	Human	[13]
Ubiquitination	NGF	HDAC6 catalyzes deacetylation of NGF, promoting ubiquitination and degradation, inhibiting the PI3K/Akt/mTOR pathway	Maintains primordial follicle quiescence; abnormal reduction in HDAC6 leads to POI (excessive follicle activation)	Mouse	[40]
	MeCP2	E3 ligase CRL4 maintains the transcription in growing oocytes by targeting MeCP2 for degradation to prevent DNA hypermethylation	Maintains primordial follicle pool	Mouse	[41]
	p27	Skp2 mediates ubiquitination and degradation of p27, relieving inhibition of the cyclin E-CDK2 complex	Promotes granulosa cell transition from G1 to S phase; Skp2 deficiency leads to POI (impaired granulosa cell proliferation)	Mouse	[42]
	SYCP3	UCHL1 removes ubiquitin chains from SYCP3, maintaining synaptonemal complex integrity	UCHL1 functional defects lead to POI (abnormal oocyte chromosome segregation, apoptosis)	Mouse	[43]
	Connexin37	FBXW7 mediates ubiquitination of Connexin37, regulating gap junction permeability	FBXW7 mutation leads to POI (impaired oocyte–granulosa cell communication)	Mouse	[44,51]
	YAP	LRRC4 promotes ubiquitination of YAP, restricting its nuclear translocation	LRRC4 deficiency leads to POI (abnormal mitochondrial fission in granulosa cells, insufficient energy supply)	Mouse	[46]
	AR	PGK1 binds Skp2 to inhibit its ubiquitination and degradation of AR, leading to AR accumulation	PCOS (increased local ovarian androgen synthesis, chronic inflammatory microenvironment)	Human	[14,47]
Acetylation	H3K9ac	Transient upregulation of H3K9ac in primordial germ cells activates germ cell-specific genes such as Ddx4 and Sycp3	Promotes primordial follicle assembly	Mammal	[54]
	α-tubulin K40ac	Ikbkap (Elp1) promotes α-tubulin K40 acetylation, maintaining spindle stability	Abnormal acetylation reduces oocyte maturation rate (spindle polarity disorder, chromosome misalignment)	Mouse	[56]
	PDK4	AMPK activation enhances SIRT1 deacetylase activity, activating PDK4 and inhibiting pyruvate dehydrogenase	Abnormalities in this pathway lead to glycolytic disorders in PCOS	Human	[33]
	SDHA	SIRT3 mediates deacetylation of SDHA, maintaining mitochondrial cristae structure and ATP production	Imbalanced acetylation reduces oocyte energy reserves and impairs embryonic development	Mouse	[57]
Lactylation	AARS2 (R199C mutation)	AARS2 mutation increases lactylation levels, inhibiting CPT2-mediated fatty acid oxidation and PDHA1-driven pyruvate entry into the tricarboxylic acid cycle	POI (excessive follicle recruitment and depletion)	Human	[62]
	Histones (H3K9la, H3K14la, H4K8la, H4K12la)	Highly expressed at the GV stage, downregulated with meiotic progression; exogenous lactate enhances their levels, upregulating oxidative phosphorylation-related genes	Promotes oocyte maturation and spindle integrity	Mouse	[64]
	H3K18la, CREB K136la	Under hCG-induced hypoxia, lactate promotes H3K18la (activating CYP11A1, STAR transcription) and CREB K136la (enhancing transcriptional activity)	Promotes progesterone synthesis during luteinization	Human	[65]
	H3K18la	H3K18la activates Ythdf2 transcription; Ythdf2 binds m^6^A-modified Ets1 mRNA to promote its degradation	Inhibits female germline stem cell proliferation, maintaining homeostasis	Mouse	[66]
SUMOylation	KLP-19 (C. elegans)	GEI-17 mediates SUMOylation of KLP-19, essential for midbivalent ring complex assembly and chromosome alignment	Ensures meiotic chromosome alignment	C. elegans	[71]
	Aurora-B (Lys207)	SUMOylation of Aurora-B at Lys207 maintains spindle stability	Mutation leads to oocyte chromosome misalignment and metaphase I arrest	Mammal	[72]
	Septin2	SUMOylation of Septin2 promotes chromosome congression in mouse oocytes	Maintains normal meiotic progression	Mouse	[73]
	PLK1	PLK1 undergoes SUMO-2/3 modification, which is reversed by SENP3, ensuring meiotic transition	Regulates oocyte maturation process	Mouse	[74]
	UBE2I	UBE2I deficiency leads to downregulation of maternal effect genes and abnormal zygotic genome activation	POI (follicle depletion)	Mouse	[77,78]
ISGylation	ADAMTS1	ISG15 promotes proteasomal degradation of ADAMTS1 via ISGylation	Inhibits ovulation; ISG15 knockout increases ovulation rate	Mouse	[84]
	CYP19A1	ISGylation upregulates CYP19A1 expression	Supports follicle health and estradiol synthesis	Pig	[83]
	USP18	USP18, as an ISG15 deconjugase, balances ISGylation	Maintains reproductive tract homeostasis; abnormalities lead to follicular atresia	Human	[85]

## Data Availability

No new data were created or analyzed in this study. Data sharing is not applicable to this article.

## Abbreviations

The following abbreviations are used in this manuscript:

PTM	Post-translational modifications
PCOS	Polycystic ovary syndrome
POI	Premature ovarian insufficiency
GCs	Premature ovarian insufficiency
FSH	Follicle-stimulating hormone
LH	Luteinizing hormone
BPA	Bisphenol A
PKA	Protein kinase A
GV	Germinal vesicle
AR	Androgen receptor
